# Development of machine learning models for predicting unfavorable functional outcomes from preoperative data in patients with chronic subdural hematomas

**DOI:** 10.1038/s41598-023-44029-2

**Published:** 2023-10-09

**Authors:** Yutaro Fuse, Yoshitaka Nagashima, Hiroshi Nishiwaki, Fumiharu Ohka, Yusuke Muramatsu, Yoshio Araki, Yusuke Nishimura, Jumpei Ienaga, Tetsuya Nagatani, Yukio Seki, Kazuhiko Watanabe, Kinji Ohno, Ryuta Saito

**Affiliations:** 1 Present Address: Department of Neurosurgery, Nagoya University Graduate School of Medicine, Nagoya, Japan; 2 Division of Neurogenetics, Nagoya University Graduate School of Medicine, Nagoya, Japan; 3 Department of Neurosurgery, Handa City Hospital, Handa, Japan; 4 Department of Neurosurgery, Japanese Red Cross Aichi Medical Center Nagoya Daini Hospital, Nagoya, Japan; 5 Present Address: Academia-Industry collaboration platform for cultivating Medical AI Leaders (AI-MAILs), Nagoya University Graduate School of Medicine, Nagoya, Japan

**Keywords:** Neurological disorders, Machine learning

## Abstract

Chronic subdural hematoma (CSDH) often causes neurological deterioration and is treated with hematoma evacuation. This study aimed to assess the feasibility of various machine learning models to preoperatively predict the functional outcome of patients with CSDH. Data were retrospectively collected from patients who underwent CSDH surgery at two institutions: one for internal validation and the other for external validation. The poor functional outcome was defined as a modified Rankin scale score of 3–6 upon hospital discharge. The unfavorable outcome was predicted using four machine learning algorithms on an internal held-out cohort (n = 188): logistic regression, support vector machine (SVM), random forest, and light gradient boosting machine. The prediction performance of these models was also validated in an external cohort (n = 99). The area under the curve of the receiver operating characteristic curve (ROC-AUC) of each machine learning-based model was found to be high in both validations (internal: 0.906–0.925, external: 0.833–0.860). In external validation, the SVM model demonstrated the highest ROC-AUC of 0.860 and accuracy of 0.919. This study revealed the potential of machine learning algorithms in predicting unfavorable outcomes at discharge among patients with CSDH undergoing burr hole surgery.

## Introduction

Chronic subdural hematoma (CSDH) is one of the most common neurosurgical conditions and is characterized by the collection of blood and fluid between the brain and the dura. CSDH is prevalent among elderly people, and its incidence is growing with the rapidly increasing aging population worldwide^1,2^. The symptoms of CSDH include headache, vomiting, speech difficulty, paralysis, confusion, and even death. Operative hematoma evacuation is the mainstay treatment of symptomatic CSDH. Generally, this condition has been considered to have a good postoperative prognosis. However, as much as 30% of patients undergoing surgery for CSDH require assistance after hospital discharge^3^. Thus, a comprehensive evaluation of the potential risks and benefits of surgery and prediction of its outcome should be conducted before initiating therapy in patients with CSDH.

Although statistical analysis has revealed the association of various preoperative factors with the postoperative functional outcome prognosis of CSDH, the predictive ability of classical statistical methods is limited. The factors correlated with poor outcomes among patients with CSDH include age^4–6^, obesity^5^, preoperative low Glasgow coma scale (GCS) score^4,6,7^, bilateral involvement^5,8^, and radiological factors, such as brain atrophy on head computed tomography (CT)^9,10^. Several studies have used multivariate regression models to predict functional outcomes^10,11^, however, their discriminative abilities were suboptimal, with area under the curve of the receiver operating characteristic curve (ROC-AUC) ranging from 0.594 to 0.624^10,12^.

Ensuring the input of appropriate variables into machine learning models and evaluating the generalization performance of the output are crucial steps for the clinical application of this technique. Owing to its ability to manage a large dataset, machine learning can be used in clinical settings that require handling of different complex variables^13^. Some studies have reported the use of machine learning models to predict the functional prognosis of patients with CSDH^10,14^. Although these models showed moderate predictive performance (accuracy of 0.727 and 0.853), their generalizability remains unclear due to the lack of external validation. Moreover, previous studies did not incorporate blood test results as input variables, which can serve as robust predictors of functional outcome. Potential outcome predictors, such as activated partial thromboplastin time (APTT), prothrombin time, and international normalized ratio on hospital admission, were recently identified^15^.

This study aimed to predict the functional prognosis in patients with CSDH before undergoing burr hole surgery by employing various machine learning algorithms and utilizing preoperative blood test results and clinical findings as inputs. The practicality and applicability of these predictive models in real-world clinical settings were assessed by performing internal and external validations.

## Methods

### Patient selection

We retrospectively collected patient data from two institutions. The patients who underwent their first burr hole surgery between June 2017 and March 2021 at Handa City Hospital were included into the internal cohort. Patients data from Japanese Red Cross Aichi Medical Center Nagoya Daini Hospital was used for external validation dataset. All patients had a preoperative diagnosis of CSDH based on a plain head CT scan or brain magnetic resonance imaging. The exclusion criteria were patients with a previous history of craniotomy, CSDH surgery, shunt surgery, and those with subdural abscess.

### Operative procedure and perioperative management

All patients underwent hematoma evacuation via single burr hole craniostomy under local anesthesia. There are three types of surgical procedures; irrigation, simple drainage, and combination. During an irrigation procedure, a flexible rubber tube was inserted into the hematoma area to drain the fluid and consequently irrigate the subdural space with normal saline solution. Simple drainage procedure requires placing a drainage tube in the subdural space. Whether to place a drainage tube after irrigation depended on the operators’ discretion. Postoperatively, patients with good postoperative neurological status were discharged from the hospital on postoperative days 3–7. Patients with speech or physical difficulties were enrolled in an in-hospital rehabilitation program. Those who could not return to home due to physical difficulties or comorbidities were transferred to a rehabilitation hospital or a long-term nursing facility.

### Data collection

Clinical findings, including age, sex, medical history, neurological symptoms, preoperative CT scan findings, drainage tube placement, and surgical information, were collected using a retrospective chart review. Baseline blood samples were routinely collected from the patients upon hospital admission for performing blood tests. The type of collected laboratory data varied at the attending neurosurgeon’s discretion. The following characteristics of hematomas were obtained from the preoperative CT: volume, thickness, and internal architecture. Hematoma volume was calculated using the method described by Won S-Y^16^. Hematoma thickness was measured on axial images. When hematomas were seen bilaterally, their volumes were summed, as were their thickness. The internal architecture of hematomas was divided into two groups based on previous reports on preoperative hematoma characteristics and recurrence^17^. One group consisted of hyperdensity, separated, laminar, gradation, or trabecular types of hematoma density on CT scan. The other was hypodensity or isodensity types. Surgeons were classified as residents (with < 5 years of training) and others (with > 5 years of experience).

The functional outcome of this study was assessed using the modified Rankin scale (mRS)^18^ at patient discharge. Originally developed for patients with stroke, the mRS can also be used for evaluating difficulties in performing daily activities in patients with CSDH^19,20^. The mRS is an ordered scale that runs from 0 (no symptoms) to 5 (severe disability) and 6 (death). The scores between 0 and 5 represent no significant disability (1), slight disability (2), moderate disability (3), and moderately severe disability (4). We defined the favorable functional outcome as having an mRS score of 0–2, as described in previous studies^3,21^. We defined an unfavorable outcome as having an mRS score of 3–6, indicating the inability to manage daily activities without assistance.

### Statistical analyses

Statistical analyses were performed to examine the association between each patient parameter and postoperative functional outcome. Univariate analysis of the dataset was conducted using R version 4.1.2 (R Foundation for Statistical Computing, Vienna, Austria) and RStudio version 2021.09.0 + 35 (Boston, Massachusetts: RStudio, Inc. 2021). The Mann–Whitney U test was used to identify perioperative variables associated with an unfavorable outcome. Fisher’s exact test was used to assess the categorical variables. P values of < 0.05 were considered significant. To prevent data contamination, the statistical analysis results were not used in constructing the machine learning prediction models described below.

### Dataset creation

Data cleaning was conducted using the dataset with R and RStudio. Demographic characteristics and laboratory data were extracted from the patients in the internal cohort as input variables for the internal dataset. Variables with missing values above 8% of all subjects were excluded as input features. Information about operative procedure and postoperative Goreisan usage were excluded from predictors because it was unavailable before surgery. Categorical variables were converted to binary features with one-hot encoding. For the external validation dataset, the same variables were collected from the patients in the external cohort.

### Machine learning and prediction model development

#### Data splitting and normalization

The internal dataset was randomly split into the training and test datasets with a split ratio of 3:1^22^. The process was performed in a stratified fashion on Python version 3.9.2. Subsequently, the variables were normalized with StandardScaler (default setting) in the Sklearn library.

#### Supervised machine learning approach

The machine learning framework was written on Python version 3.9.2 using standard machine learning libraries. The framework comprised three steps (feature selection, data balancing, and training of classifiers) using the training dataset.

Variables were selected using two methods: the filter and wrapper methods. As one of the standard filter methods, univariate feature selection with SelectPercentile was conducted using sklearn.feature_selection package. SelectPercentile is a method used to rank each variable and to extract variables according to the percentile of the highest scores. Percentiles of 5, 10, 15, 20, 50, and 100 were applied. Conversely, recursive feature elimination, one of the popular wrapper-type feature selection methods, was used. It fits a model (Random forest) and eliminates the least important variables until the specified number of features is reached^23^. The recursive feature elimination process was cross-validated using the training dataset to identify the optimal number of features utilized to build a machine learning model.

The outcome variable of the dataset was imbalanced (favorable, 67%: unfavorable, 33%); therefore, two data balancing techniques were applied. One is undersampling of most data under the condensed nearest neighbor rule. The other is the upsampling of the minority of the data. A commonly used approach—the Synthetic Minority Over-sampling Technique (SMOTE)—was applied^24^.

Four machine learning classifiers (logistic regression, support vector machine (SVM), random forest, and light gradient boosting machine (light GBM)) were trained to predict unfavorable outcomes after CSDH surgery. The rationale behind choosing these four models^25^ was rooted in the aim of evaluating representative machine learning models that are based on substantially varied architectural principles. This selection was guided by factors such as the models' interpretability, capacity to handle missing data, and their potential to deliver effective performance within the framework of our clinical applications. Their hyperparameters were optimized to give the largest area under the curve of the receiver operating characteristic curve (ROC-AUC) via grid search with fivefold cross-validation. The hyperparameter combinations that were explored through grid search are documented in Supplementary Table S1.

Logistic regression is a traditional statistical method used for dichotomous classification, and it is a technique used to apply regression algorithms to classification tasks using an S curve to provide input values between 0 and 1. Logistic regression is now considered as one of the basic machine learning algorithms.

SVM is another machine learning method used for binary classification. Its name is derived from support vectors, which are the points closest to the line or hyperplane dividing the dataset. The distance between the support vectors and line/hyperplane is called the margin. SVM creates a boundary that maximizes the margin, thereby separating the two classes.

Random forest, a combination of decision trees, is a robust machine learning method invented in 2001^26^. A large number of decision trees are created via random sampling using datasets. Subsequently, the majority vote of the predicted results for each tree is taken to determine the final predictive value. Random forest has a lower risk of overfitting than a single decision tree because it uses multiple trees to prevent the influence of variance or bias.

Light GBM is a relatively novel type of gradient boosting decision tree algorithm developed in 2017^27^. A gradient-boosting decision tree builds decision trees (weak learners) one-by-one to minimize the error of the previous model. Light GBM is a modified version of the original gradient-boosting decision tree model by reducing computation time while maintaining prediction accuracy.

### Evaluation of the machine learning models

After hyperparameters were fixed, the machine learning models were tested using the test data of the internal dataset. This evaluation was conducted independently of the training process. ROC curve analysis was conducted to evaluate the discrimination ability of the models. The accuracy, sensitivity, specificity, and f1 score of each model were compared at the optimal cutoff point as set by Youden’s index^28^. The model exhibiting the highest ROC-AUC in the evaluation metrics was selected as the best-performing model, and its associated preprocessing methods and hyperparameter settings were meticulously recorded. In terms of the interpretation of algorithm predictions, values of standardized beta coefficients of the logistic regression were calculated. The coefficients describe the size and direction of the association between the predictor and outcome. Moreover, the feature importance of the random forest and light GBMs were evaluated. Feature importance is expressed as a score assigned to features based on their contribution to the prediction model. The score was computed based on the total impurity reduction of splits (Gini importance). The best-performing model was validated using the external validation dataset for each type of the four machine learning algorithms.

### Ethical statement

This retrospective study is approved by the Ethics Review Committee of Nagoya University Graduate School of Medicine (2021-0442). Since this study was noninvasive, the Ethics Review Committee of Nagoya University Graduate School of Medicine approved that the requirement for written informed consent from patients was waived, but the opt-out method was adopted in accordance with the Japanese ethics guidelines. A public notice regarding this study was given on the websites of Handa City Hospital and Japanese Red Cross Aichi Medical Center Nagoya Daini Hospital. This research was completed in accordance with the Declaration of Helsinki as revised in 2013.

## Results

### Overview of the development of the internal dataset

**Figure 1:**
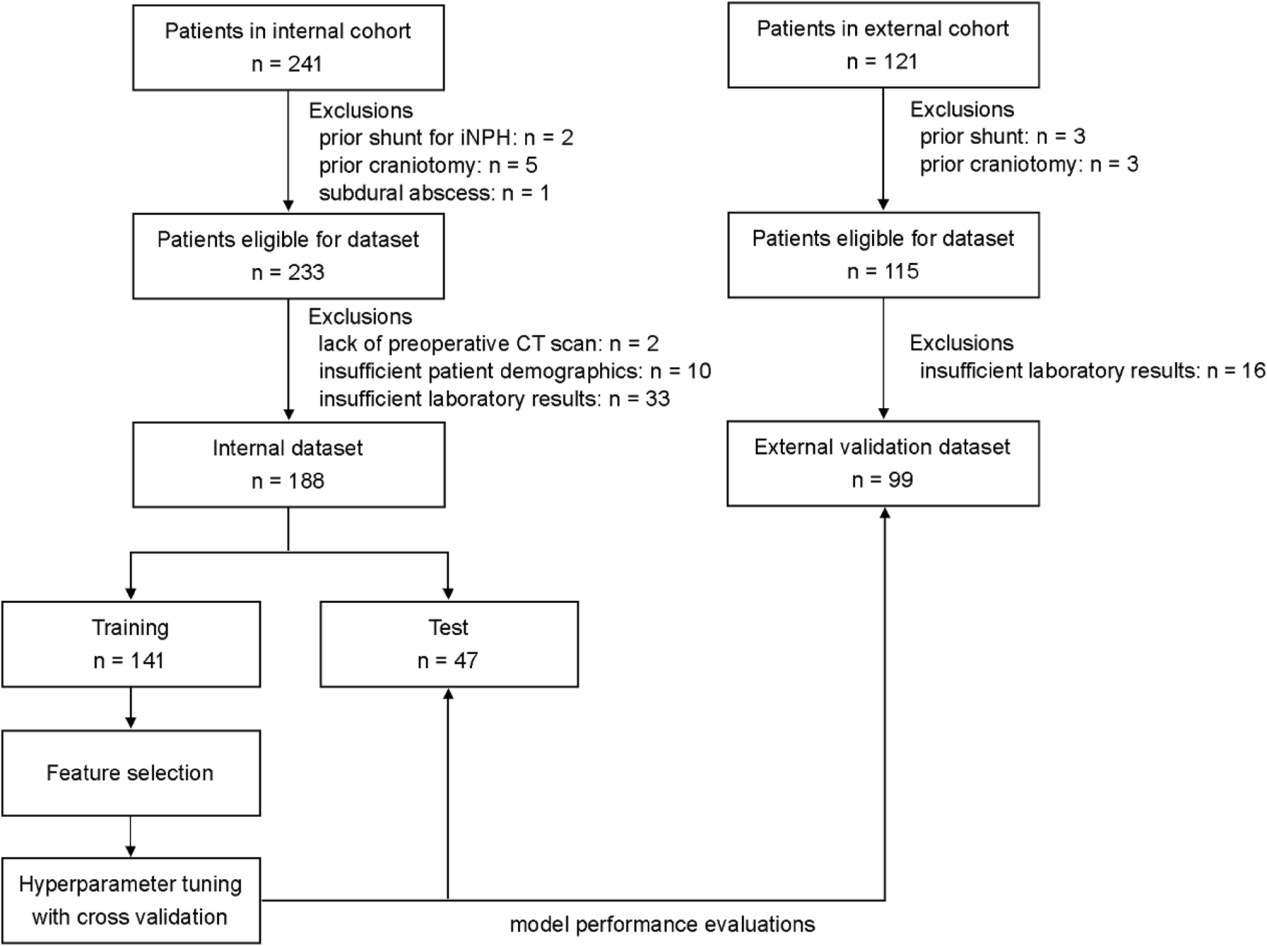
Flow chart of patient selection and establishment of the machine learning models. iNPH, idiopathic normal pressure hydrocephalus; CT, computed tomography.

In total, 241 patients with CSDH who experienced their first burr hole surgery were eligible for the internal cohort of this study. Exclusion criteria were met in eight patients: prior shunt for idiopathic normal pressure hydrocephalus (n = 2), prior craniotomy (n = 5), and subdural abscess (n = 1). Data from the remaining 233 patients were analyzed to determine input variables for the creation of the prediction model. A total of 52 variables comprising 19 categorical and 33 continuous features were extracted as predictors (Tables 1, 2). Two of the 233 patients were excluded for the lack of preoperative CT scans. Moreover, ten patients were excluded because of unavailable accurate patient background, and 33 were excluded for missing variables of the laboratory results. The remaining 188 patients were finally included in this study to build the internal dataset. The training dataset comprised 141 patients, whereas the test dataset comprised 47 patients. The flowchart of this procedure is depicted in Fig. 1 (left).

**Table 1:** Characteristics and treatment of patients assessed via univariate analyses.

Baseline characteristics and treatment	The internal cohort			The external cohort		
	Functional outcome at discharge		p-value^2^	Functional outcome at discharge		p-value^2^
	Favorable N = 126^1^	Unfavorable N = 62^1^		Favorable N = 84^1^	Unfavorable N = 15^1^	
Age	77 (12)	85 (6)	** < 0.001**	78 (10)	83 (9)	0.073
Sex			0.2			0.8
Female	36 (29%)	24 (39%)		27 (32%)	6 (40%)	
Male	90 (71%)	38 (61%)		57 (68%)	9 (60%)	
Body mass index	22.0 (3.2)	20.4 (3.2)	**0.003**	22.0 (3.3)	21.5 (3.6)	0.6
GCS on admission			**0.002**			**0.030**
14–15	90 (71%)	29 (47%)		73 (87%)	9 (60%)	
3–13	36 (29%)	33 (53%)		11 (13%)	6 (40%)	
Hemiparesis	79 (63%)	44 (71%)	0.3	68 (81%)	15 (100%)	0.14
Speech difficulty	8 (6.3%)	13 (21%)	**0.006**	16 (19%)	5 (33%)	0.4
Preceding trauma events	76 (60%)	33 (53%)	0.4	49 (58%)	10 (67%)	0.7
Hemisphere			0.8			0.9
Bilateral	25 (20%)	10 (16%)		25 (30%)	4 (27%)	
Left	56 (44%)	28 (45%)		33 (39%)	7 (47%)	
Right	45 (36%)	24 (39%)		26 (31%)	4 (27%)	
Hypertension	62 (49%)	39 (63%)	0.11	38 (45%)	10 (67%)	0.2
Diabetes mellitus	28 (22%)	11 (18%)	0.6	16 (19%)	5 (33%)	0.4
Dyslipidemia	35 (28%)	15 (24%)	0.7	26 (31%)	3 (20%)	0.6
Chronic kidney disease	36 (29%)	18 (29%)	> 0.9	8 (9.5%)	0 (0%)	0.5
Ischemic stroke	10 (7.9%)	9 (15%)	0.3	9 (11%)	5 (33%)	0.056
Cardiovascular disease	7 (5.6%)	6 (9.7%)	0.5	9 (11%)	1 (6.7%)	> 0.9
Liver disease, alcohol abuse	7 (5.6%)	1 (1.6%)	0.4	6 (7.1%)	0 (0%)	0.6
Anticoagulant use	6 (4.8%)	6 (9.7%)	0.3	2 (2.4%)	1 (6.7%)	> 0.9
Antiplatelet use	19 (15%)	16 (26%)	0.11	17 (20%)	4 (27%)	0.8
mRS before onset			** < 0.001**			** < 0.001**
0–2	115 (91%)	30 (48%)		81 (96%)	6 (40%)	
3–6	11 (8.7%)	32 (52%)		3 (3.6%)	9 (60%)	
Hematoma density on preoperative CT			0.3			0.8
Hyperdensity, separated, laminar, gradation, trabecular	95 (75%)	52 (84%)		73 (87%)	14 (93%)	
Hypodensity, isodensity	31 (25%)	10 (16%)		11 (13%)	1 (7%)	
Preoperative hematoma volume	148 (118, 194)	163 (130, 194)	0.13	146 (123, 196)	138 (120, 169)	0.6
Preoperative hematoma thickness	2.50 (2.10, 2.98)	2.45 (2.00, 3.10)	0.9	2.60 (2.10, 3.10)	2.40 (2.00, 2.80)	0.4
Midline shift	8.0 (4.9, 11.1)	8.4 (5.2, 11.1)	0.7	6.9 (4.5, 10.7)	5.8 (3.4, 7.8)	0.12

^1^Data are presented as the mean ± standard deviation (SD), the median (interquartile range: IQR), or the number (%).

^2^Pearson's Chi-squared test; Mann–Whitney U test.

Significant values are in bold.

**Table 2:** Preoperative laboratory data evaluated via univariate analyses.

Laboratory data on admission	The internal cohort			The external cohort		
	Functional outcome at discharge		p-value^2^	Functional outcome at discharge		p-value^2^
	Favorable, N = 126^1^	Unfavorable, N = 62^1^		Favorable, N = 84^1^	Unfavorable, N = 15^1^	
Hemoglobin (g/dL)	13.00 (12.03, 14.47)	11.80 (10.50, 12.50)	** < 0.001**	12.85 (11.70, 14.00)	12.00 (11.20, 12.85)	0.053
RBC (*10^6^/μL)	416 (379, 454)	376 (350, 417)	** < 0.001**	406 (365, 444)	387 (356, 403)	0.11
WBC (*10^3^/μL)	6.80 (5.90, 7.68)	6.40 (5.00, 8.17)	0.6	6.75 (5.70, 8.10)	8.90 (5.85, 10.95)	0.065
Platelet (*10^3^/μL)	20 (17, 25)	21 (17, 28)	0.5	19.9 (16.5, 23.4)	22.1 (19.4, 27.7)	0.11
MCHC (g/dL)	34.45 (33.50, 34.98)	33.60 (32.50, 34.10)	** < 0.001**	34.25 (33.60, 34.80)	34.00 (33.70, 34.50)	0.4
MCH (pg)	31.40 (30.30, 32.88)	30.80 (29.15, 32.08)	**0.015**	31.45 (30.37, 32.52)	31.80 (30.00, 32.25)	0.8
MCV (fL)	91.4 (88.0, 95.0)	92.0 (87.7, 94.5)	0.8	91.7 (88.6, 94.8)	92.2 (89.2, 93.7)	> 0.9
Hematocrit (%)	38.0 (35.3, 41.5)	34.9 (31.6, 37.1)	** < 0.001**	37.7 (34.3, 40.5)	34.9 (32.0, 37.4)	0.057
Basophil (%)	0.30 (0.20, 0.50)	0.20 (0.12, 0.30)	** < 0.001**	0.50 (0.40, 0.80)	0.30 (0.20, 0.45)	**0.006**
Eosinophil (%)	1.50 (0.52, 2.85)	1.65 (0.50, 2.85)	> 0.9	1.60 (0.60, 2.87)	0.20 (0.10, 0.70)	** < 0.001**
Monocyte (%)	5.45 (4.62, 6.40)	5.95 (4.53, 6.90)	0.13	7.75 (6.57, 9.20)	7.50 (5.80, 10.70)	> 0.9
Lymphocyte (%)	20 (13, 25)	19 (12, 24)	0.14	19 (14, 26)	15 (8, 17)	**0.004**
Neutrophil (%)	72 (65, 79)	72 (65, 81)	0.5	70 (63, 77)	77 (72, 84)	**0.013**
PT-INR	1.00 (0.96, 1.08)	1.05 (1.01, 1.14)	** < 0.001**	1.03 (0.99, 1.06)	1.02 (0.99, 1.10)	0.5
APTT (sec)	31.0 (29.3, 34.1)	30.9 (29.3, 33.5)	0.7	25.50 (24.25, 27.55)	26.20 (23.80, 28.45)	0.6
BUN (mg/dL)	18 (13, 21)	18 (14, 28)	0.2	17 (13, 21)	18 (15, 22)	0.6
Creatinine (mg/dL)	0.78 (0.62, 1.01)	0.77 (0.60, 1.04)	> 0.9	0.80 (0.66, 1.05)	0.75 (0.54, 1.15)	0.5
eGFR (ml/min/1.7)	68 (52, 86)	66 (47, 84)	0.3	67 (47, 76)	59 (45, 86)	0.8
ALT (U/L)	16 (12, 23)	13 (10, 21)	**0.027**	14 (10, 20)	14 (12, 18)	0.7
AST (U/L)	22 (18, 28)	22 (18, 29)	0.8	25 (21, 32)	26 (24, 34)	0.4
Total bilirubin (mg/dL)	0.70 (0.50, 1.00)	0.60 (0.40, 0.78)	**0.030**	0.69(0.48, 0.90)	0.59(0.46, 0.82)	0.6
Total protein (g/dL)	6.90 (6.70, 7.30)	6.80 (6.43, 7.10)	**0.035**	7.06 (6.73, 7.41)	7.21 (6.97, 7.56)	0.2
Albumin (g/dL)	4.00 (3.73, 4.30)	3.65 (3.20, 3.90)	** < 0.001**	4.02 (3.75, 4.25)	3.45 (2.96, 4.03)	**0.002**
Albumin/globulin ratio	1.37 (1.21, 1.56)	1.17 (1.03, 1.31)	** < 0.001**	1.32 (1.10, 1.47)	0.89 (0.69, 1.15)	** < 0.001**
Glucose (mg/dL)	118 (102, 142)	117 (100, 146)	0.8	114 (103, 141)	141 (118, 171)	**0.044**
CK (U/L)	114 (74, 175)	101 (54, 254)	0.5	103 (65, 179)	121 (66, 155)	> 0.9
Na (mmol/L)	140.0 (138.0, 142.0)	140.4 (137.0, 142.0)	0.8	138.5 (137.0, 140.0)	137.0 (133.0, 139.0)	0.090
K (mmol/L)	4.00 (3.80, 4.20)	4.00 (3.62, 4.38)	> 0.9	3.90 (3.60, 4.30)	4.00 (3.80, 4.10)	0.9
Cl (mmol/L)	104.0 (102.2, 106.0)	105.0 (100.2, 107.0)	0.9	105.0 (103.0, 107.0)	104.0 (101.0, 105.5)	0.3

RBC, red blood cell; WBC, white blood cell; MCHC, mean corpuscular hemoglobin concentration; MCH, mean corpuscular hemoglobin; MCV, mean corpuscular volume; PT-INR, prothrombin time-international normalized ratio; APTT, activated partial thromboplastin time; BUN, blood urea nitrogen; eGFR, estimated glomerular filtration rate; ALT, alanine aminotransferase; AST, aspartate aminotransferase; CK, creatine kinase; mRS, modified Rankin scale.

^1^Median (Interquartile range).

^2^Mann-Whitney U test.

Significant values are in bold.

### Baseline characteristics of the included patients

The baseline characteristics of patients with the evaluation via univariate analyses are shown in Table 1. The mean age of the patients upon hospital admission was 79 years. As previously reported^4,5,7^, the unfavorable outcome group was significantly older than the favorable outcome group (p < 0.001; mean age is 85 or 77 years, respectively). Moreover, patients in the unfavorable outcome group were more likely to experience low GCS scores (≤ 13) upon admission (53%) than those in the favorable outcome group (29%; p = 0.002). The ratio of mRS 0–2 before the onset was 91% in the favorable outcome group and 48% in the unfavorable outcome group (p < 0.001). Other significantly different characteristics between the two groups were BMI (p = 0.003) and speech difficulty upon admission (p = 0.006).

### Preoperative laboratory data of the study patients

Preoperative laboratory data on admission with the evaluation via univariate analyses are presented in Table 2. The unfavorable outcome group had a significantly lower serum albumin level of 3.65 g/dL, compared to the favorable outcome group (4.00 g/dL; p < 0.001). Similarly, the albumin/globulin ratio was lower in the unfavorable outcome group (unfavorable: 1.17, favorable: 1.37; p < 0.001). Moreover, hemoglobin level was significantly lower in the unfavorable outcome group (unfavorable: 11.80 g/dL, favorable: 13.00 g/dL; p < 0.001). A hematocrit test showed that the unfavorable outcome group had a lower percentage of red blood cells (34.9%) than the other group (38.0%; p < 0.001). Other components of laboratory data significantly lower in the unfavorable outcome group were red blood cell counts (p < 0.001), mean corpuscular hemoglobin concentration (MCHC; p < 0.001), mean corpuscular hemoglobin (MCH; p = 0.015), the percentage that basophils account for white blood cell count (p < 0.001), alanine aminotransferase (p = 0.027), total bilirubin (p = 0.030), and total protein levels (p = 0.035). Meanwhile, the unfavorable outcome group had a significantly higher INR than the favorable outcome group (p < 0.001).

### The operative procedure, surgeon expertise, and postoperative Goreisan usage

Within the internal cohort of patients, a total of 98 individuals (52%) underwent irrigation and drainage procedures, while the remaining underwent irrigation exclusively. Within the group with favorable outcomes, 56% of patients underwent irrigation and drainage, whereas this was the case for 44% in the unfavorable outcome group (p = 0.13). Regarding the surgeon's expertise, a majority of patients, 154 (82%), were operated on by residents, with no statistically significant difference in distribution between the two outcome groups (favorable: 89% vs. unfavorable: 79%, p = 0.13). Ninety-nine patients (79%) in the favorable outcome group and 41 patients (66%) in the unfavorable outcome group employed Goreisan post-surgery, with no statistically significant difference between the two groups (p = 0.10).

### Optimal preprocessing methods and hyperparameter configurations for the machine learning algorithms

The prime logistic regression model was established using features within the upper 20th percentile (11 features) as determined by the SelectPercentile method, without employing data balancing techniques. Among the chosen 11 features, four originated from clinical findings, while seven were derived from laboratory data. The optimal light GBM model also hinged upon the same 11 features. The model utilized the undersampling technique through the condensed nearest neighbor rule. Conversely, the best SVM model was developed using the features in the top 10th percentile, encompassing six attributes (two from clinical findings and four from laboratory data), without necessitating any data balancing techniques. The top-performing random forest model emerged from the features in the top 15th percentile, designated by the SelectPercentile method. This optimal random forest model adopted the oversampling technique via SMOTE. The preprocessing methods and integrated hyperparameters in the top-performing machine learning models are shown in Table 3. An overview of the entire set of combinations can be found in Supplementary Table S2.

**Table 3:** Preprocessing methods and hyperparameters incorporated into the top-performing machine learning models.

Models	Feature selection methods	Number of selected features	Data balancing technique	Hyperparameters
Logistic regression	SelectPercentile: 20	11	None	'max_iter' : 5000, 'class_weight' : {1:1}
SVM	SelectPercentile: 10	6	None	'C': 0.001, 'gamma': 0.001, 'probability':True
Random forest	SelectPercentile: 15	8	Oversampling (SMOTE)	'criterion': 'gini', 'n_estimators': 1000, 'max_features': 'sqrt', 'min_samples_leaf': 2, 'min_samples_split': 5, 'max_depth': 200, 'class_weight': None
Light GBM	SelectPercentile: 20	11	Undersampling (CNN)	'bagging_fraction': 0.8, 'bagging_freq': 1, 'feature_fraction': 0.8, 'learning_rate': 0.01, 'num_leaves': 7

SVM, support vector machine; light GBM, light gradient boosting machine; ROC-AUC, area under the curve of the receiver operating characteristic curve; SelectPercentile: 20, top 20 percent of the features were selected by the SelectPercentile method; SMOTE, the Synthetic Minority Over-sampling Technique; CNN, the condensed nearest neighbor.

### Comparison of the performances of the models

**Figure 2:**
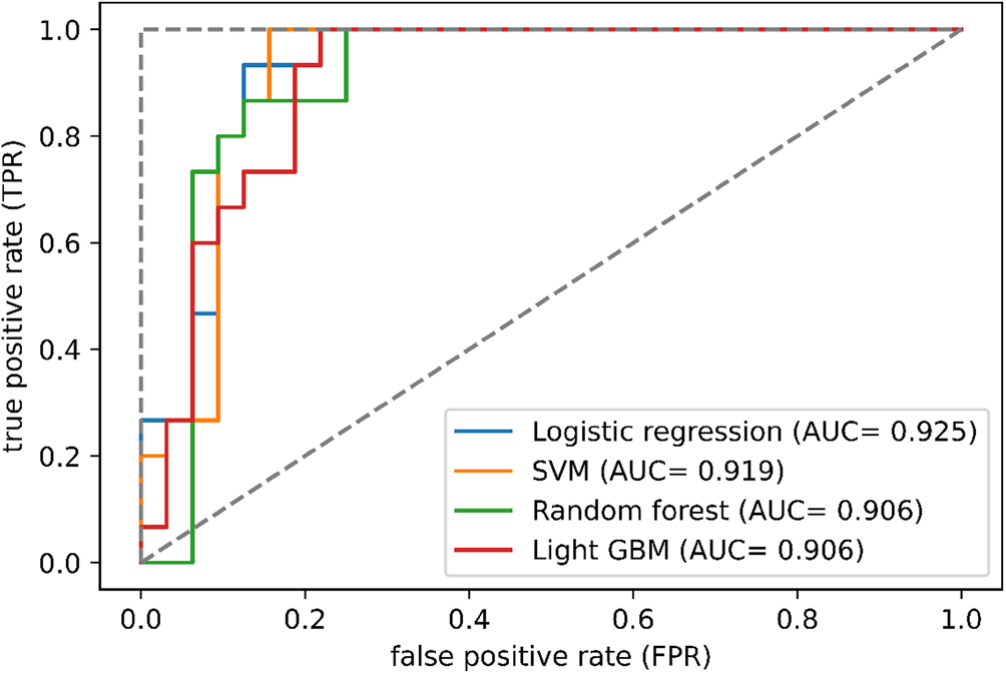
Receiver operating characteristic curves of the four machine learning algorithms on the internal dataset. AUC, area under the curve; SVM, support vector machine; light GBM, light gradient boosting machine.

The accuracy, sensitivity, specificity, F1 score, and ROC-AUC of the four prediction models that were trained on the optimally selected features are shown in Table 4. The performance metrics of the four machine learning algorithms trained on all features without selection are presented in Supplementary Table S3. The prediction accuracy scores of all four models with optimal feature selection were higher than the scores of the models trained for all features. The highest accuracy score of 0.894 was observed in the logistic regression and SVM models (Table 4). The accuracies in the random forest and light GBM models were 0.830 and 0.851, respectively. Among the prediction models, the logistic regression model demonstrated the highest specificity of 0.875. In contrast, the other three models demonstrated the highest sensitivity of 1.000. Among the evaluated machine learning models, F1 score was the highest in the SVM model. ROC curves of the four machine learning algorithms are illustrated in Fig. 2. The largest ROC-AUC of 0.925 was demonstrated in the logistic regression model. ROC-AUC values of the SVM, random forest, and light GBM models were 0.919, 0.906, and 0.906, respectively.

**Table 4:** Comparison of the predictive abilities of the four machine learning algorithms on the test dataset.

Models	Accuracy	Sensitivity	Specificity	f1 score	ROC-AUC
Logistic regression	0.894	0.933	0.875	0.848	0.925
SVM	0.894	1.000	0.844	0.857	0.919
Random forest	0.830	1.000	0.750	0.789	0.906
Light GBM	0.851	1.000	0.781	0.811	0.906

SVM, support vector machine; light GBM, light gradient boosting machine; ROC-AUC, area under the curve of the receiver operating characteristic curve.

### Evaluation of input variable importance for machine learning model development

**Figure 3:**
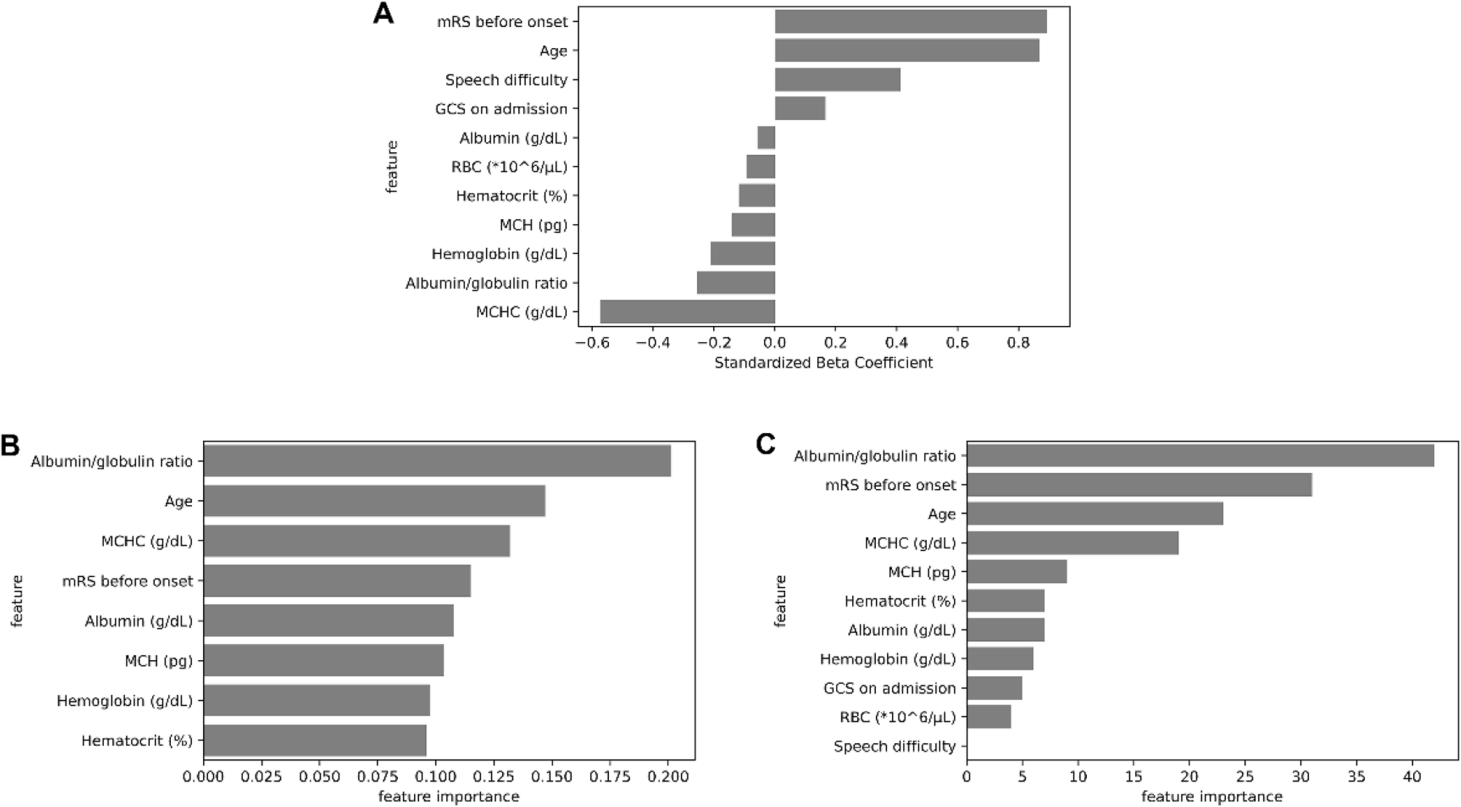
Selected features and their importance. (**A**) Standard beta coefficient of the features selected in the logistic regression model. (**B**) Importance of the features selected in the random forest model. (**C**) Importance of the features selected in the light gradient boosting machine model. mRS, modified Rankin scale; GCS, Glasgow coma scale; RBC, red blood cell; MCH, mean corpuscular hemoglobin; MCHC, mean corpuscular hemoglobin concentration.

The value of the standardized beta coefficient of the logistic regression model is illustrated in Fig. 3A. The highest value of the beta coefficient was 0.894 in the feature “mRS before onset,” followed by 0.870 in “Age”. In contrast, all the selected features of the laboratory data had negative coefficients. MCHC (− 0.572) and albumin/globulin ratio (− 0.255) were the top two among the seven variables. The feature importances of the random forest and light GBM models are depicted in Fig. 3B and C, respectively. Both machine learning models ranked albumin/globulin ratio, mRS before onset, age, and MCHC among the top four essential features.

### External validation of the machine learning models

A total of 121 consecutive patients with CSDH who had not experienced prior burr hole surgery were eligible for the external validation cohort. Six patients were excluded due to prior craniotomy or shunt surgery. Sixteen patients were excluded due to missing laboratory data, and the remaining 99 patients were included in the analysis (Fig. 1, right). A favorable outcome was observed in 84 patients (85%), whereas an unfavorable outcome was noted in 15% of patients. All input variables from demographic and laboratory data are illustrated with univariate analysis in Tables 1 and 2. Of the baseline characteristics, low GCS scores upon admission (favorable, 13%, vs. unfavorable, 40%; p = 0.030) and ratio of mRS 0–2 before the onset (96% vs. 40%; p < 0.001) were statistically different between the favorable and unfavorable outcome groups. Unlike the internal cohort, BMI and the presence of speech difficulty during onset were not significantly different (p = 0.6 and p = 0.4, respectively). Preoperative laboratory data on admission shows that the serum albumin level (favorable, 4.02, vs. unfavorable, 3.45 g/dL; p = 0.002) and albumin/globulin ratio (1.32 vs. 0.89; p < 0.001) were significantly different between the two groups. Serum glucose level was significantly higher in the unfavorable outcome group (favorable, 114 mg/dL; unfavorable, 141 mg/dL; p = 0.044), which was inconsistent with the internal cohort.

**Figure 4:**
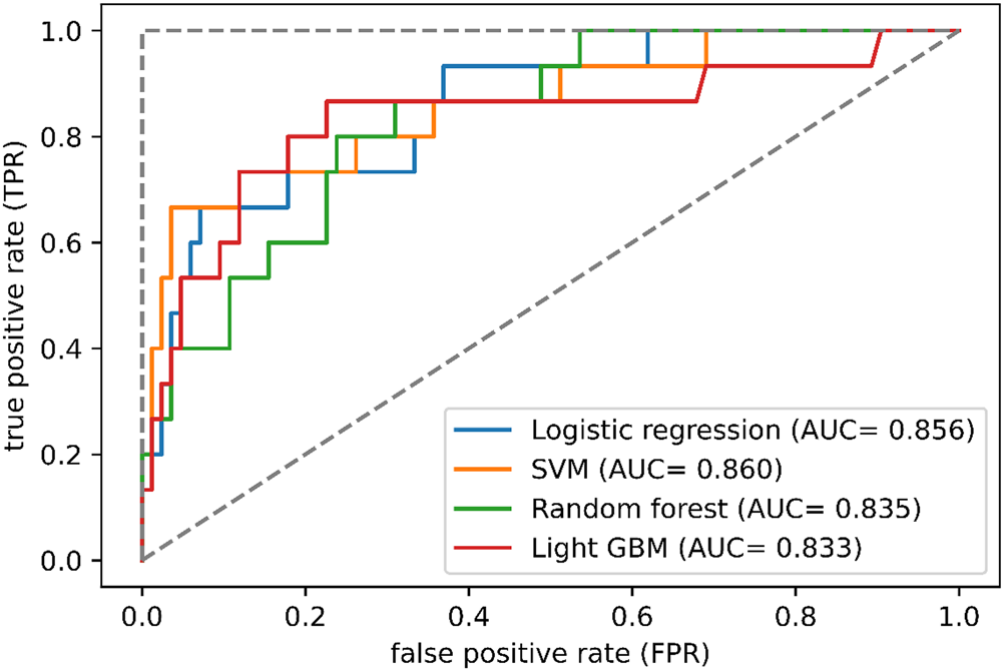
Receiver operating curves of the four machine learning algorithms in the external cohort. AUC, area under the curve; SVM, support vector machine; light GBM, light gradient boosting machine.

The performance metrics of each machine learning model on the external validation dataset are shown in Table 5. Among the prediction models, the SVM model demonstrated the highest accuracy of 0.919, followed by the logistic regression (0.889), light GBM (0.788), and random forest (0.768) models. The light GBM model yielded the highest sensitivity of 0.867, tailed by the random forest of 0.800 and logistic regression and SVM of 0.667. The highest ROC-AUC value of 0.860 was observed in the SVM model. The ROC-AUC values of the logistic regression, random forest and light GBM models were 0.856, 0.835, and 0.833, respectively. The ROC curves of the proposed machine learning models are summarized in Fig. 4.

**Table 5:** The predictive performance of the four machine learning algorithms in external cohort.

Models	Accuracy	Sensitivity	Specificity	f1 score	ROC-AUC
Logistic regression	0.889	0.667	0.929	0.645	0.856
SVM	0.919	0.667	0.964	0.714	0.860
Random forest	0.768	0.800	0.762	0.511	0.835
Light GBM	0.788	0.867	0.774	0.553	0.833

SVM, support vector machine; light GBM, light gradient boosting machine; ROC-AUC, area under the curve of the receiver operating characteristic curve.

## Discussion

The objective of this study was to develop various machine learning models that integrate preoperative blood test results and clinical findings to predict postoperative functional outcome of CSDH patients who underwent burr hole surgery. This study compared multiple machine learning algorithms, including logistic regression, SVM, random forest, and light GBM. The ROC-AUCs of all machine learning approaches in the internal cohort dataset were high (0.906–0.925). Adding blood test results into candidate predictors and selecting essential ones from them contributed to the increase in the prediction performance of the machine learning models. The cross-validation method was used to identify the optimal number of input features and the best hyperparameters of each machine learning model. To strengthen its reproductivity and reliability, the prediction models were validated in an external cohort. The ROC-AUCs of these models showed satisfactory performance, ranging from 0.833 to 0.860 on the external cohort.

This study showed that postoperative functional outcome at hospital discharge can be efficiently predicted in patients with CSDH. Although surgical intervention is the primary treatment for most patients with CSDH, some patients require prolonged rehabilitation or transfer to nursing-care facilities and some even experience mortality even after the successful evacuation of hematomas. The frequency of patients with CSDH experiencing these unfavorable outcomes is increasing^3^. Thus, an early indication of postoperative functional status is helpful for health workers in deciding treatment, rehabilitation programs, and the most appropriate discharge destinations for patients with CSDH. Therefore, the accurately prediction of prognosis based on the preoperative information has great clinical value.

Within the realm of preprocessing techniques, feature selection notably contributed to an enhancement in machine learning performance in this study. In contrast, the outcomes derived from data balancing methods varied across different models. The elimination of input variables irrelevant to the target variable stands as a pivotal step in constructing a robust machine learning model^23^. As demonstrated in Supplementary Table S2, the improvement in predictive performance for machine learning models was more noticeable when utilizing the filter method compared to the wrapper method. This difference might be because the wrapper method typically demands a substantial volume of samples to build a robust model, which might not have been fully available in our study. Regarding data balancing, both logistic regression and SVM exhibited superior results without implementing data balancing techniques. Oversampling carries the risk of overfitting, while undersampling harbors the risk of essential features being compromised. These potential drawbacks may have had a more significant impact on their performance. Conversely, random forest and light GBM, characterized by their resilience to overfitting, are models that can seamlessly operate even when confronted with missing variables. As a result, these models may have effectively addressed the challenges inherent in data balancing and adeptly capitalized on its benefits.

The strength of this study is the comprehensive acquisition of preoperative clinically available factors that were entered into the machine learning models. Previous postoperative prognostic models for chronic subdural hematoma relied on patients’ background factors, physical findings, and imaging results^10,11,14^. Moreover, to the best of our knowledge, this is the first machine learning model designed for predicting prognosis in CSDH using comprehensive blood test data. Even after applying a feature selection method on the input variables, seven predictors derived from laboratory results were found to be relevant factors to predict unfavorable outcomes, with serum MCHC levels showing the highest coefficient value in the logistic regression model. Furthermore, the feature importance in the random forest and light GBM models demonstrated that preoperative blood test parameters, including albumin/globulin ratio, were vital in the construction of both models. Our results also showed that poor mRS score before onset, old age, speech difficulty, and GCS score upon admission were also important to predict unfavorable functional outcomes. These findings were consistent with previous studies^4,5^ and our clinical experience. Our study revealed that patients’ background, clinical findings, and blood test results were useful predictors of postoperative functional prognosis in CSDH patients.

Another strength of this study is that we performed external validation to confirm the generalizability of the machine learning models. Validation using a single dataset cannot exclude the possibility of overfitting, which limits the applicability of the model to new data. Previous studies using machine learning models for outcome prediction in patients with CSDH lacked external validation^10,14^, making it difficult to ascertain the actual generalization performance of the models. Another scoring model for predicting postoperative functional outcome designed by Kwon et al. showed an excellent ROC-AUC score of 0.948 in internal validation^11^; however, the score significantly decreased to 0.624 in external validation^12^. This indicated that the generalizability of the model may be limited. Contrarily, our machine learning models maintained a relatively high ROC-AUC score ranging from 0.833 to 0.860 within the external validation cohort of a distinct institution. Nevertheless, it is imperative to acknowledge that this performance was coupled with a substantial decrease in sensitivity. This observation implies that the adaptability and applicability of this study’s models could potentially be circumscribed within certain contextual scenarios.

Previous studies have suggested that the volume and type of CSDH and midline shift are associated with postoperative recurrence^17,29^; however, they were not significantly related to unfavorable outcomes at discharge in this study. Anticoagulant and antiplatelet use have also been associated with postoperative recurrence^30^, though they were not selected as essential features in the presented prediction models. Thus, the present study suggests that unfavorable outcomes at discharge and postoperative recurrence for CSDH are distinct predictors. These results indicate that the crucial characteristics vary depending on the value desired to be predicted. Therefore, as we have done in this study, providing a large number of candidate features and selecting the important features are essential in improving the predictive performance of machine learning models.

This study used machine learning algorithms that differed from the models previously used to predict the functional prognosis of CSDH. Prior studies have used artificial neural network (ANN) and classification and regression tree (CART) methods^10,14^. ANN is a network of connected neurons, and its architecture is based on at least three layers: input, hidden, and output. Abouzari et al. demonstrated that ANN outperformed logistic regression in predicting the discharge outcomes of patients with CSDH^10^. However, in our study, ANN was not used as it typically requires a large number of cases to generate accurate outcomes^31^ and its black-box nature decimates the interpretability of the input variables. CART is a machine learning method used to build a decision tree based on input variables. Rovlias et al. employed CART to predict the functional outcomes at 3 months after CSDH surgery^14^. We used random forest and light GBM algorithms instead of CART as the decision tree–based method. The random forest and light GBM algorithms can create multiple decision trees, which increases the accuracy and reduces the overfitting of data compared with using a single tree.

This study has developed machine learning prediction models employing diverse architectures, and their clinical applicability should be predicated upon their respective attributes. To begin, the logistic regression model emerges as notably interpretable, courtesy of its derived coefficients. This inherent interpretability imparts the advantage of expounding to patients the rationale underlying the outcomes computed by the machine learning model. SVM slightly trails behind logistic regression in terms of interpretability; nevertheless, the SVM model devised in this study possesses the merit of relying solely on six variables. This attribute makes the model well-suited for providing rapid decision support to physicians, as predictions can be efficiently generated by inputting a concise set of variables. Distinctly, random forests and light GBMs demonstrate the ability to make predictions even when confronted with missing data, endowing them the advantage of accommodating patients with limited availability of clinical information for collection.

The current study had several limitations. First, it was a retrospective study, which inevitably inherited relatively significant variance and biases; therefore, cross-validation was conducted during feature selection and hyperparameter tuning to improve the robustness of machine learning models. In addition, to eliminate the possibility of data contamination, validation processes using the test dataset and external cohort were conducted independently from the training process. Second, while we employed four well-established machine learning models, it is important to acknowledge the existence of other widely recognized prediction modeling algorithms, such as Lasso regression and k-nearest neighbors methods. Furthermore, the landscape of machine learning algorithms is continuously evolving. As algorithms with superior interpretability and enhanced predictive capabilities continue to emerge, we remain committed to harnessing them to further enhance accuracy in our endeavors.

This study aimed to predict the postoperative functional outcome of patients with CSDH who underwent burr hole surgery via the integration of a comprehensive set of preoperative clinical factors, including blood tests, into machine learning models. The proposed machine learning models effectively predicted the physical function prognosis at discharge. Moreover, the generalization performance of the models was confirmed using external validation. Overall, our study findings suggested that integrating preoperative clinical factors into machine learning models could accurately predict postoperative functional outcomes in patients with CSDH.

### Supplementary Information

Supplementary Tables.

## Data Availability

The datasets used and/or analyzed during the current study are available from the corresponding author on reasonable request. The code of the prediction models in this study is available at https://github.com/yutarofuse/CSDH_mRS_prediction.
