# Supplementary material for: Development of machine learning models for predicting unfavorable functional outcomes from preoperative data in patients with chronic subdural hematomas
Source: Sci Rep. 2023 Oct 9;13:16997. doi: 10.1038/s41598-023-44029-2 (PMC10562441; doi:10.1038/s41598-023-44029-2)
Supplement: Supplementary file 1 — Supplementary Tables. [file 41598_2023_44029_MOESM1_ESM.pdf]

Supplementary Table S1. Hyperparameter combinations explored through grid search

models	hyperparameter combinations
logistic regression	{'max_iter' : [5000], 'class_weight' : ['balanced', '{{1:1}}']}
SVM	{'C': [0.001, 0.01, 0.1, 1, 10, 100], 'gamma' : [0.001, 0.01, 0.1, 1, 10, 100], 'probability':[True]}
random forest	{'criterion' : ['gini'], 'n_estimators': [1000], 'max_features': ['sqrt'], 'min_samples_leaf': [2, 5, 10], 'min_samples_split': [2, 5], 'max_depth': [7, 63, 200], 'class_weight': ['balanced', None]}
light GBM	{'num_leaves': [7, 15, 31], 'learning_rate': [0.1, 0.01, 0.005], 'feature_fraction': [0.5, 0.8], 'bagging_fraction': [0.8], 'bagging_freq': [1, 3]}

SVM, support vector machine; light GBM, light gradient boosting machine; ROC-AUC, area under the curve of the receiver operating characteristic curve.

Supplementary Table S2. Comparison of the ROC-AUC scores of the four machine learning models with feature selection and over/undersampling techniques.

ML models	Feature selection methods	Oversampling/ Undersampling	N of selected features	ROC-AUC
LR	SelectPercentile: 5	-	3	0.896
LR	SelectPercentile: 10	-	6	0.917
LR	SelectPercentile: 15	-	8	0.917
**LR**	**SelectPercentile: 20**	**-**	**11**	**0.925***
LR	SelectPercentile: 50	-	26	0.848
LR	SelectPercentile: 100	-	53	0.850
LR	SelectPercentile: 5	SMOTE	3	0.898
LR	SelectPercentile: 10	SMOTE	6	0.913
LR	SelectPercentile: 15	SMOTE	8	0.910
LR	SelectPercentile: 20	SMOTE	11	0.910
LR	SelectPercentile: 50	SMOTE	26	0.819
LR	SelectPercentile: 100	SMOTE	53	0.833
LR	SelectPercentile: 5	CNN	3	0.896
LR	SelectPercentile: 10	CNN	6	0.898
LR	SelectPercentile: 15	CNN	8	0.894
LR	SelectPercentile: 20	CNN	11	0.896
LR	SelectPercentile: 50	CNN	26	0.833
LR	SelectPercentile: 100	CNN	53	0.752
LR	RFECV	-	15	0.892
LR	RFECV	SMOTE	18	0.875
LR	RFECV	CNN	26	0.815
SVM	SelectPercentile: 5	-	3	0.898
**SVM**	**SelectPercentile: 10**	**-**	**6**	**0.919***
SVM	SelectPercentile: 15	-	8	0.908
SVM	SelectPercentile: 20	-	11	0.917

Supplementary Table S2. Comparison of the ROC-AUC scores of the four machine learning models with feature selection and over/undersampling techniques.

ML models	Feature selection methods	Oversampling/ Undersampling	N of selected features	ROC-AUC
SVM	SelectPercentile: 50	-	26	0.867
SVM	SelectPercentile: 100	-	53	0.815
SVM	SelectPercentile: 5	SMOTE	3	0.898
SVM	SelectPercentile: 10	SMOTE	6	0.715
SVM	SelectPercentile: 15	SMOTE	8	0.819
SVM	SelectPercentile: 20	SMOTE	11	0.900
SVM	SelectPercentile: 50	SMOTE	26	0.627
SVM	SelectPercentile: 100	SMOTE	53	0.713
SVM	SelectPercentile: 5	CNN	3	0.894
SVM	SelectPercentile: 10	CNN	6	0.917
SVM	SelectPercentile: 15	CNN	8	0.403
SVM	SelectPercentile: 20	CNN	11	0.892
SVM	SelectPercentile: 50	CNN	26	0.850
SVM	SelectPercentile: 100	CNN	53	0.306
SVM	RFECV	-	22	0.879
SVM	RFECV	SMOTE	7	0.900
SVM	RFECV	CNN	18	0.863
RF	SelectPercentile: 5	-	3	0.871
RF	SelectPercentile: 10	-	6	0.883
RF	SelectPercentile: 15	-	8	0.865
RF	SelectPercentile: 20	-	11	0.883
RF	SelectPercentile: 50	-	26	0.831
RF	SelectPercentile: 100	-	53	0.854
RF	SelectPercentile: 5	SMOTE	3	0.838
RF	SelectPercentile: 10	SMOTE	6	0.865
**RF**	**SelectPercentile: 15**	**SMOTE**	**8**	**0.906***
RF	SelectPercentile: 20	SMOTE	11	0.894
RF	SelectPercentile: 50	SMOTE	26	0.800
RF	SelectPercentile: 100	SMOTE	53	0.810
RF	SelectPercentile: 5	CNN	3	0.881
RF	SelectPercentile: 10	CNN	6	0.877
RF	SelectPercentile: 15	CNN	8	0.888
RF	SelectPercentile: 20	CNN	11	0.883
RF	SelectPercentile: 50	CNN	26	0.844
RF	SelectPercentile: 100	CNN	53	0.817
RF	RFECV	-	19	0.840
RF	RFECV	SMOTE	12	0.729
RF	RFECV	CNN	18	0.792

Supplementary Table S2. Comparison of the ROC-AUC scores of the four machine learning models with feature selection and over/undersampling techniques.

ML models	Feature selection methods	Oversampling/ Undersampling	N of selected features	ROC-AUC
LGBM	SelectPercentile: 5	-	3	0.893
LGBM	SelectPercentile: 10	-	6	0.896
LGBM	SelectPercentile: 15	-	8	0.883
LGBM	SelectPercentile: 20	-	11	0.888
LGBM	SelectPercentile: 50	-	26	0.850
LGBM	SelectPercentile: 100	-	53	0.792
LGBM	SelectPercentile: 5	SMOTE	3	0.806
LGBM	SelectPercentile: 10	SMOTE	6	0.860
LGBM	SelectPercentile: 15	SMOTE	8	0.865
LGBM	SelectPercentile: 20	SMOTE	11	0.840
LGBM	SelectPercentile: 50	SMOTE	26	0.744
LGBM	SelectPercentile: 100	SMOTE	53	0.767
LGBM	SelectPercentile: 5	CNN	3	0.886
LGBM	SelectPercentile: 10	CNN	6	0.898
LGBM	SelectPercentile: 15	CNN	8	0.850
**LGBM**	**SelectPercentile: 20**	**CNN**	**11**	**0.906***
LGBM	SelectPercentile: 50	CNN	26	0.840
LGBM	SelectPercentile: 100	CNN	53	0.865
LGBM	RFECV	-	19	0.856
LGBM	RFECV	SMOTE	19	0.742
LGBM	RFECV	CNN	17	0.796

ML, machine learning; LR, logistic regression; SVM, support vector machine; RF, random forest; LGBM, light gradient boosting machine; RFECV, recursive feature elimination with cross-validation; SMOTE, the Synthetic Minority Over-sampling Technique; CNN, Condensed Nearest Neighbor; N, number; ROC-AUC, area under the curve of the receiver operating characteristic curve.

*Best-scored models; their performance metrics are shown in Table 3.

**Models without feature selection; their performance metrics are depicted in Supplementary Table S3.

Supplementary Table S3. Comparison of the predictive abilities of the four machine learning algorithms by using all 52 input variables.

models	accuracy	sensitivity	specificity	f1 score	ROC-AUC
logistic regression	0.830	0.933	0.781	0.778	0.850
SVM	0.830	0.733	0.875	0.733	0.733
random forest	0.787	0.800	0.781	0.706	0.810
light GBM	0.830	0.867	0.813	0.765	0.865

SVM, support vector machine; light GBM, light gradient boosting machine; ROC-AUC, area under the curve of the receiver operating characteristic curve.
